# Brain Networks of Novelty-Driven Involuntary and Cued Voluntary Auditory Attention Shifting

**DOI:** 10.1371/journal.pone.0044062

**Published:** 2012-08-28

**Authors:** Samantha Huang, John W. Belliveau, Chinmayi Tengshe, Jyrki Ahveninen

**Affiliations:** 1 Athinoula A. Martinos Center for Biomedical Imaging, Department of Radiology, Massachusetts General Hospital, Harvard Medical School, Charlestown, Massachusetts, United States of America; 2 Harvard-MIT Division of Health Sciences and Technology, Cambridge, Massachusetts, United States of America; Baycrest Hospital, Canada

## Abstract

In everyday life, we need a capacity to flexibly shift attention between alternative sound sources. However, relatively little work has been done to elucidate the mechanisms of attention shifting in the auditory domain. Here, we used a mixed event-related/sparse-sampling fMRI approach to investigate this essential cognitive function. In each 10-sec trial, subjects were instructed to wait for an auditory “cue” signaling the location where a subsequent “target” sound was likely to be presented. The target was occasionally replaced by an unexpected “novel” sound in the uncued ear, to trigger involuntary attention shifting. To maximize the attention effects, cues, targets, and novels were embedded within dichotic 800-Hz vs. 1500-Hz pure-tone “standard” trains. The sound of clustered fMRI acquisition (starting at *t* = 7.82 sec) served as a controlled trial-end signal. Our approach revealed notable activation differences between the conditions. Cued voluntary attention shifting activated the superior intra­­parietal sulcus (IPS), whereas novelty-triggered involuntary orienting activated the inferior IPS and certain subareas of the precuneus. Clearly more widespread activations were observed during voluntary than involuntary orienting in the premotor cortex, including the frontal eye fields. Moreover, we found ­evidence for a frontoinsular-cingular attentional control network, consisting of the anterior insula, inferior frontal cortex, and medial frontal cortices, which were activated during both target discrimination and voluntary attention shifting. Finally, novels and targets activated much wider areas of superior temporal auditory cortices than shifting cues.

## Introduction

The human brain can process only a limited amount of auditory information at a time. Attention shifting is constantly needed to allow redirecting of our focus to detect the most relevant sounds amongst noise. Such shifts can be triggered top-down, for example, to voluntarily shift the focus based on our goals and interests (an endogenous process), or bottom-up, when a potentially interesting unexpected sound involuntarily captures our attention (an exogenous process). The exact neuronal mechanisms controlling these two modes of auditory attention shifting are not, however, fully clear.

Previous neuroimaging studies in this field, which have mainly concentrated on the visuo-spatial domain, suggest that shifting of attention activates a network of brain areas including dorsolateral prefrontal cortex, premotor, medial frontal areas, and the posterior parietal cortex. On the basis of these studies, it has been proposed that separate dorsal (superior parietal lobule, SPL, intraparietal sulcus, IPS, frontal eye fields, FEF) and ventral (right temporal-parietal junction, ventral frontal cortex/anterior insula) attention systems underlie voluntary *vs*. involuntary attention shifting processes, respectively [1]–[4]. However, the distinction between dorsal and ventral attention systems is still under debate, as a number of visual [5]–[9] fMRI studies have failed to find fully segregated neural systems subserving endogenous and exogenous spatial orienting.

Despite the critical role that auditory information processing plays in human communication, a much smaller number of fMRI studies have been conducted to investigate voluntary attention shifting in the auditory, compared to the visual modality. The results obtained in different studies have not been fully consistent either. For example, a recent study [10] suggested that automatic orienting, compared to controlled orienting, is associated with greater activations in several frontal and parietal regions, while others [11], [12] have reported increased activations in the posterior parietal cortex associated with top-down control of attention shifting. Inconsistencies like this are, obviously, in part related to differences in the experimental designs. At the same time, previous studies on auditory attention shifting have seldom controlled for potential biases caused by the acoustical scanner noise, which can mask the auditory stimuli and modulate the BOLD response in auditory [13] or even non-auditory cortices [14].

Resolving trade-offs related to acoustical scanner noise might be particularly essential for studies on involuntary attention shifting, an area of research that has been much more intensively investigated [15]–[18] than voluntary auditory orienting. Notably, this line of research has been, almost exclusively, based on methods such as MEG and EEG that are not biased by factors such as scanner noise. According to these studies, involuntary attention is triggered by an automatic change-detection process in superior temporal auditory cortices, as reflected by the mismatch negativity (MMN) response. This mismatch detection process is then followed by a sequence of brain events associated with attentional orienting and conscious detection of the sound change in extra-auditory association areas, which potentially involve dorsolateral prefrontal cortices [19]–[22], anterior cingulate regions [23], and/or inferior frontal gyrus [24]–[26]. However, the relative contributions of auditory areas and other regions contributing to automatic change detection and involuntary orienting are not yet fully clear.

Another factor that has received relatively little attention in classic orienting studies is the role of the anterior insula in attention shifting. Accumulating evidence suggests that the anterior insula, one of the structures originally proposed to be associated with the ventral attention system [3], may actually play an important role in voluntary cognitive control [27]–[30] and perceptual decision making [31]. A number of recent imaging studies on visual and auditory task switching, auditory working memory, and auditory attention have reported activations in the anterior insula [32]–[36]. The anterior insula has, consequently, been conjectured to contribute to the switching of attention [17] and to the related top-down interference resolution processes [37]. It has also been recently suggested that the anterior insula constitutes a supramodal region that controls the orienting of attention [36]. However, the exact role of this region in top-down aspects of auditory attention shifting needs further investigation.

In the present study, we investigated voluntary and involuntary attention shifting using a paradigm modified from classic visuospatial cued orienting [38], auditory-spatial selective attention [39], [40], and auditory involuntary attention-shifting [16], [41] designs. Stimulus-driven orienting was triggered by unexpected novel sounds, a strategy that has been well documented to produce strong event-related potential responses and behavioral distraction effects related to involuntary attention shifting [16], [42]. Biases related to acoustical scanner noises were controlled by using a mixed design, which combined event-related and sparse sampling approaches.

## Results

### Behavioral Data

**Figure 1:**
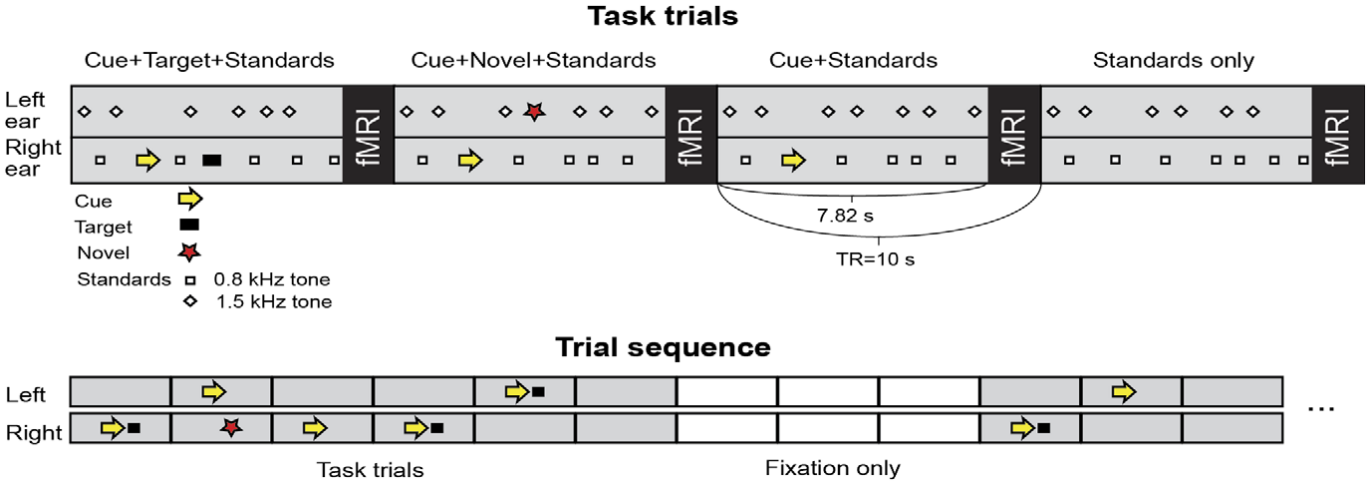
Task and stimuli. In each 10-sec trial, subjects were instructed to wait for a cue in the ear where a subsequent target was likely to appear, and to press a button as quickly as possible after hearing the target. The cues and targets were embedded within dichotic trains of pure-tone standards. Novel sounds, which occasionally occurred opposite to the cued ear, were to be ignored. All stimuli were presented during a 7.82-sec period preceding the fMRI acquisition. Subjects were informed that the sound of the scanner ended the trial (i.e., the scanner noise was a controlled trial-end signal). The proportions of the active trials were as follows: the cue followed by the target (“Cue+Target+Standards”, 40%), the cue but no target (“Cue+Standards”, 20%), the cue followed by a novel (“Cue+Novel+Standards”, 20%), and standard-stimulation only (“Standards”, 20%). A “mixed” trial-sequence design was used. That is, each period of 6 random-order active trials was followed by a block of 3 silent baseline trials (for example, to allow for quality control of within-subject auditory activations). Finally, withineach trial, the stimulus-onset asynchrony (SOA) was jittered to mitigate expectancy confounds such as omission responses. The overall inter-stimulus interval was 530 ms (during the period between the scans; corresponding to 1.06 sec within one ear, resulting in mean SOA 1.1 sec/ear).

The present auditory task design (**Figure 1**) was modified from classic visual attention shifting [38] and auditory selective attention [39] paradigms (see **Materials and Methods**)**.** During fMRI acquisition, subjects were instructed to detect a monaural harmonic target sound, which was embedded within trains of high- and low-pitch pure tones presented asynchronously to their left and right ears respectively. Outliers were defined as responses longer or slower than two standard deviations of the average reaction times within each run and counted as misses in the final behavioral data. One subject was excluded because of an inability to perform the task. In the final dataset (N = 18, 11 females, age 19–28 years), the average hit rate was 90.2±7.9% and the reaction time was 495±48 ms. The mean±SD false alarm rate, as calculated from Cue+Standards and Cue+Novel+Standards trials, was 1.2±1.5%.

To verify the beneficial effect of cues in directing attention to subsequent targets, we conducted a separate behavioral control analysis (*N* = 10, 4 females, age 22–43 years). The result demonstrated that spatial cueing significantly (*t*(9) = −4.17, *P*<0.01) speeded-up target discrimination, as compared to “invalidly cued” trials where the target occurred in the ear opposite of the cue (mean±SD reaction times 463±68 *vs*. 555±105 ms to validly *vs*. invalidly cued targets, respectively). To make tentative inferences of cueing benefits during fMRI, the data from this behavioral group were also compared to the main fMRI group’s performance during the fMRI session. There were no significant differences in the reaction times to validly cued targets during the control or main experiment. The reaction times to the invalidly cued targets during the behavioral control experiment were, however, significantly longer (*t*(26) = 2.24, *P*<0.05) than the reaction times during fMRI to validly cued targets, suggesting that subjects may have been benefiting from the spatial cueing also during the fMRI experiment.

### fMRI Results

**Figure 2:**
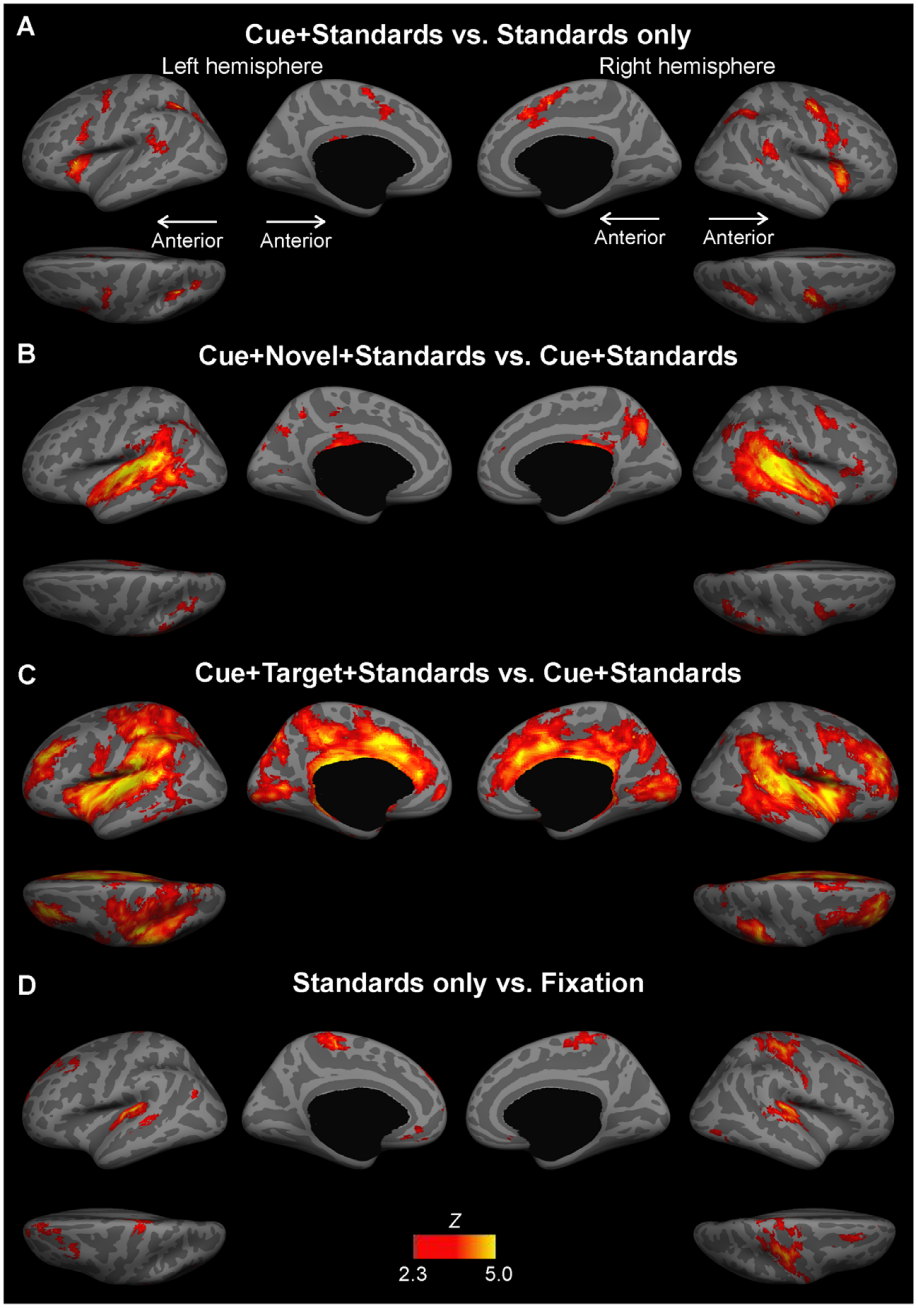
The main contrasts of the group fMRI analyses. **A.** The contrast between Cue+Standards vs. Standards only, presumably reflecting cued voluntary attention shifting. While the strongest activation focus emerged in the anterior insula, significant activations were also found in the bilateral PMC/FEF, mSFC (including pre-SMA), paracingulate, aMCC, dPCC, pSTG, PT, STS, and IPS. **B.** The contrast between Cue+Novel+Standards vs. Cue+Standards, presumably reflecting novelty-triggered involuntary attention shifting. Significant activations were found in the right PMC/FEF, MFC, pas triangularis, orbital, pregenual ACC, subparietal regions, left cuneus, bilateral posterior insula, pMCC, dPCC, Heschl’s gyrus, aSTG, pSTG, PT, STS, MTG, ITG, TPJ, IPC, IPS, and precuneus. **C.** The contrast between condition Cue+Target+Standards and Cue+Standards, presumably reflecting target discrimination. Significant activations were found in the bilateral SFC, DLPFC, PMC, IFC, orbital ACC, subgenual ACC, pregenual ACC, aMCC, pMCC, dPCC, pars marginalis, Heschl’s gyrus, aSTG, pSTG, PT, STS, MTG, ITG, TPJ, SPL, SMG, AG, IPS, subparietal sulcus, precuneus, parieto-occipital sulcus, cuneus, calcarine, and lingual gyrus. **D.** The “baseline” contrast between condition Standards only and Fixation. In addition to the primary auditory cortex, significant activations were observed in several frontal and parietal regions. The *Z*-statistic images were thresholded at *Z* >2.3 with a GRF corrected cluster significance threshold of *P*<0.05.

**Figure 2** shows activations associated with the main contrasts, presumed to reflect cued attention shifting, novelty-triggered attention shifting, and target discrimination. The anatomical areas associated with these activations have been identified in **Tables 1** **,** **2** **,** **3** based on the parcellation included the FreeSurfer package [43]. Our approach revealed notable activation differences between cued attention shifting, novelty-trigger attention shifting, and target discrimination. The specific contrasts that were utilized to determine these effects have been described below.

**Table 1:** Brain regions activated in the Cue+standards vs. Standards only contrast.

Regions of Interest	Left hemisphere							Right hemisphere						
Frontal-cingulate-insular Regions	x	Y	z	PSC	Zmax	Zc1b	Zc2b	x	y	z	PSC	Zmax	Zc1b	Zc2b
Superior frontal gyrus	−4	−2	62	0.59	3.6	1.52	−2.12	6	20	48	0.62	4.55	3.31	−3.44
Inferior frontal gyrus - pars opercularis	−36	18	10	0.51	3.85	3.64	−3.56	38	20	10	0.53	3.68	3.35	−2.87
Inferior frontal sulcus	−44	14	26	0.4	3.54	2.77	−1.47	36	18	22	0.47	3.15	2.4	−2.01
Precentral gyrus	−44	−4	48	0.69	3.28	3.24	−1.04	44	−4	50	0.98	5.05	4.15	−1.73
Inferior part of the precentral sulcus	−40	8	24	0.48	4.11	1.35	−2.85	34	6	32	0.54	3.92	1.91	−1.35
Central sulcus								40	−8	52	0.43	3.7	2.96	−0.44
Superior part of the precentral sulcus	−32	−8	50	0.33	3.74	3.08	−2.75	42	−4	48	0.43	4.57	3.84	−1.47
Middle-anterior part of the cingulate gyrus & sulcus	−8	10	46	0.31	3.12	1.33	−3.26	10	12	52	0.39	4.31	1.6	−2.44
Middle-posterior part of the cingulate gyrus & sulcus								10	4	52	0.33	4.36	2.11	−2.56
Superior segment of the circular sulcus of the insula	−30	28	8	0.45	5.18	3.25	−3.38	30	24	10	0.49	4.68	2.75	−3.27
Anterior insula - short insular gyri	−30	22	6	0.36	3.96	3.75	−3.5	34	20	0	0.42	4.39	3.52	−3.68
Anterior segment of the circular sulcus of the insula	−28	28	0	0.32	4.2	2.72	−3.86	30	24	0	0.43	4.02	3.31	−2.89
**Temporal Regions**														
Lateral superior temporal gyrus	−62	−44	14	0.6	3.5	1.99	−0.36	64	−36	12	0.58	3.41	2.81	1.8
Superior temporal gyrus - planum temporale	−52	−42	20	0.48	3.41	2.49	−0.06	64	−32	16	0.4	3.09	2.79	2.32
Superior temporal sulcus	−56	−46	12	0.41	3.05	2.95	−0.6	46	−44	12	0.46	3.47	1.53	−1.42
**Parietal Regions**														
Inferior parietal - supramarginal gyrus								60	−40	16	0.5	3.58	2.79	0.46
Inferior parietal - angular gyrus	−28	−68	42	0.43	3.43	1.22	−3.18	34	−64	44	0.43	3.41	1.49	−1.93
Sulcus intermedius primus of Jensen								60	−38	16	0.38	3.58	2.93	0.9
intraparietal sulcus and transverse parietal sulci	−30	−56	38	0.49	5.1	1.88	−3.46	32	−60	44	0.47	3.63	2.04	−2.2
**Interlobar Regions**														
Posterior ramus of the lateral sulcus	−50	−42	22	0.35	3.28	2.88	0.01							
Pericallosal sulcus	−4	−30	28	0.4	3.02	−1.16	−3.99							

Note: x,y,z coordinates in units of mm; PSC: percent signal change of the group average of each subject’s maximum percent signal change in the region; Zmax: the Z-score of the peak voxel of activation within the local cluster; Zc1b: the Z-score of the 1st condition cue+standards vs. baseline; Zc2b:the Z-score of the 2nd condition standards vs. baseline.

**Table 2:** Brain regions activated in the Cue+Novel+Standards vs. Cue+Standards contrast.

Regions of Interest	Left hemisphere							Right hemisphere						
Frontal-cingulate-insular Regions	x	y	z	PSC	Zmax	Zc1b	Zc2b	x	y	z	PSC	Zmax	Zc1b	Zc2b
Middel frontal gyrus								40	12	46	0.65	3.03	1.92	−1.5
Inferior frontal gyrus - pars triangularis								52	28	4	0.49	3.25	0.5	−4.01
Precentral gyrus								44	2	46	0.65	3.49	3.52	2.31
Inferior part of the precentral sulcus								42	2	40	0.41	3.36	3.54	1.72
Subcentral gyrus (central operculum) and sulci	−48	−18	16	0.5	3.63	1.89	−2.46	54	−8	12	0.57	3.77	1.12	−1.24
Orbital sulcus								28	38	−6	0.69	2.63	1.86	−1.64
Superior part of the precentral sulcus								44	0	44	0.33	3.35	3.65	2.77
Middle-posterior part of the cingulate gyrus and sulcus	−4	−20	32	0.36	3	1.19	−3.18							
Posterior-dorsal part of the cingulate gyrus	−4	−26	32	0.39	3.2	1.15	−2.4	6	−26	30	0.38	3.16	2.99	−1.27
Marginal branch of the cingulate sulcus	−16	−32	42	0.28	2.98	−1.46	−3.2							
Posterior insula	−40	0	−16	0.6	4.01	2.95	−2.16	38	4	−18	0.85	4.05	2.65	−2.6
Superior segment of the circular sulcus of the insula								32	26	10	0.3	3.02	4.03	3.14
Anterior insula								40	4	−4	0.28	3.02	−0.72	−2.9
Inferior segment of the circular sulcus of the insula	−44	−22	4	1.09	5.46	5.27	2.79	46	−18	−4	0.93	5.44	4.21	1.35
**Temporal Regions**														
Heschl’s gyrus	−46	−20	6	1.09	5.84	4.98	0.71	54	−6	2	0.99	5.56	2.98	−1.12
Transverse temporal sulcus	−40	−30	8	1.15	5.43	5.56	3.72	50	−28	10	1.19	5.53	5.83	3.19
Lateral superior temporal gyrus (STG)	−64	−40	12	1.67	5.82	4.92	3.33	64	−34	6	1.71	6.29	6.01	2.4
Superior temporal gyrus - planum polare	−50	−6	−6	0.93	5.2	5.22	0.36	50	−6	−6	0.9	5.18	4.08	−0.01
Superior temporal gyrus - planum temporale (PT)	−56	−32	10	1.47	5.49	5.78	3.11	60	−30	14	1.33	5.92	5.05	3.93
Superior temporal sulcus	−56	−50	6	0.95	4.72	3.14	0.69	52	−36	10	0.91	5.72	5	1.96
Middle temporal gyrus	−58	−54	8	0.78	5.12	2.91	0.22	62	−38	−2	1	4.65	2.42	−1.36
Inferior temporal gyrus	−56	−58	−10	0.64	3.67	−1.66	−3.52	56	−46	−12	0.82	3.59	−0.22	−3.1
Inferior temporal sulcus	−44	−60	6	0.45	3.56	1.5	−1.68	58	−42	−10	0.48	3.15	0.09	−2.95
Medial occipito-temporal gyrus - parahippocampal gyrus	−18	−32	−8	0.77	2.92	−0.33	−2.94	16	−34	−6	0.81	2.92	0.4	−2.3
**Parietal Regions**														
Inferior parietal - supramarginal gyrus (SMG)	−60	−46	20	0.75	4.99	2.82	1	64	−32	14	1.1	5.12	4.55	3.65
Inferior parietal - angular gyrus (AG)	−58	−50	20	0.58	4.12	2.31	0.59	54	−46	26	0.49	4	3.5	−0.11
Sulcus intermedius primus of Jensen	−52	−50	30	0.34	3.23	2	−1.87	58	−42	16	0.64	4.84	3.58	2.39
intraparietal sulcus (IPS) and transverse parietal sulci	−30	−66	38	0.38	3.22	2.68	0.81	34	−66	34	0.34	3.02	1.98	−0.28
Subparietal sulcus								12	−54	44	0.35	3.19	0.08	−3.68
Precuneus	−6	−52	48	0.54	3.4	−0.65	−3.23	8	−66	34	0.58	4	3.79	−0.13
**Occipital Regions**														
Middle occipital gyrus								34	−70	34	0.35	2.92	1.2	−1.21
Cuneus	−6	−80	32	0.54	3.15	−0.77	−2.78							
Anterior occipital sulcus	−44	−62	6	0.3	3.44	1.66	−1.43	48	−60	8	0.29	3	1.7	−1.26
**Interlobar Regions**														
Horizontal ramus of the anterior segment of the lateral fissure								46	30	4	0.32	3.13	0.49	−1.89
Vertical ramus of the anterior segment of the lateral sulcus								40	26	10	0.25	3.18	1.98	−0.78
Posterior ramus of the lateral sulcus	−46	−34	10	1.01	5.53	5.01	1.44	50	−30	10	0.82	5.77	5.83	2.79
Pericallosal sulcus	−2	−28	28	0.54	3.73	2.69	−1.5	4	−24	28	0.45	4.15	3.42	−1.66
Parieto-occipital sulcus	−10	−70	40	0.4	3.27	1.93	−0.37	10	−64	34	0.48	3.99	3.51	0.37

Note: x,y,z coordinates in units of mm; PSC: percent signal change of the group average of each subject’s maximum percent signal change in the region; Zmax: the Z-score of the peak voxel of activation within the local cluster; Zc1b: the Z-score of the 1^st^ condition cue+novel+standards vs. baseline; Zc2b: the Z-score of the 2^nd^ condition cue+standards vs. baseline.

**Table 3:** Brain regions activated in the Cue+Target+Standards vs. Cue+Standards contrast.

Regions of Interest	Left hemisphere							Right hemisphere						
Frontal-cingulate-insular Regions	x	y	z	PSC	Zmax	Zc1b	Zc2b	x	y	z	PSC	Zmax	Zc1b	Zc2b
Superior frontal gyrus	−6	32	36	1.3	4.15	1.37	−1.72	20	50	30	1.18	3.72	0.12	−3.55
Superior frontal sulcus	−26	38	30	0.39	4.74	2.64	−2.05	26	40	32	0.39	4.28	2.81	−1.33
Middel frontal gyrus	−34	38	30	0.89	5.21	4.13	−1	38	36	32	0.88	5.01	4.11	−1.37
Middle frontal sulcus	−34	38	30	0.47	5.21	4.13	−1	36	36	32	0.5	4.99	4.65	−0.71
Inferior frontal gyrus - pars orbitalis	−30	32	−2	0.39	2.97	2.58	−1.07							
Inferior frontal gyrus - pars opercularis	−54	2	8	0.84	4.97	5.15	−1.89	46	6	6	1	5.58	4.61	−0.29
Inferior frontal gyrus - pars triangularis								48	38	2	0.7	4.23	1.08	−2.53
Inferior frontal sulcus	−38	42	12	0.45	4.43	2.67	−0.2	38	40	18	0.56	4.58	3.5	0.3
Precentral gyrus	−38	−20	58	1.83	4.19	3.15	−3.39	56	8	12	0.99	5.26	2.5	1.73
Superior part of the precentral sulcus	−24	−12	58	0.47	3.37	2.89	−2.09	44	0	46	0.47	3.23	3.65	2.82
Inferior part of the precentral sulcus	−54	4	12	0.51	3.73	3.96	−0.98	50	8	10	0.48	3.6	3.78	1.47
Central sulcus	−36	−20	54	0.99	4.16	2.6	−4.32							
Subcentral gyrus (central operculum) and sulci	−42	−8	14	1.21	5.97	4.64	−3.75	50	−6	10	0.95	4.23	3.11	−1.29
Orbital gyri	−22	10	−14	0.95	3.43	1.84	−4.03	24	10	−14	1.14	4.14	2.7	−3.17
Lateral orbital sulcus								40	42	2	0.42	4	1.25	−1.3
Orbital sulcus	−24	38	−8	0.52	3.73	2.23	−2.45	24	36	−8	0.7	3.34	2.53	−2.62
Olfactory sulcus	−16	8	−14	0.72	4.13	2.46	−4.08	20	8	−14	0.87	3.63	2.85	−2.61
Transverse frontopolar gyri and sulci								22	56	2	0.88	3.5	1.25	−0.82
Fronto-marginal gyrus (of Wernicke) and sulcus	−22	52	0	0.77	3.22	3.12	−1.75	20	56	−6	0.76	4.64	2.86	−1.9
Anterior part of the cingulate gyrus and sulcus	−6	36	14	0.47	5.21	3.05	−3.9	6	32	24	0.57	4.96	4.48	−0.82
Middle-anterior part of the cingulate gyrus and sulcus	−8	6	38	1.09	5.55	5.39	−2.02	10	26	28	0.91	5.5	4.62	0.74
Middle-posterior part of the cingulate gyrus and sulcus	−8	6	38	1.15	5.55	5.39	−2.02	6	−14	32	0.79	5.03	3.7	−2.35
Posterior-dorsal part of the cingulate gyrus	−2	−32	30	0.7	4.91	3.66	−1.99	8	−44	24	0.65	4.48	3.48	−2.57
Posteror-ventral part of the cingulate gyrus	−10	−42	6	0.47	4.17	0.94	−3.5	16	−38	2	0.55	4.16	3.09	−3.11
Marginal branch of the cingulate sulcus	−8	−34	44	0.59	5.05	2.42	−3.95	14	−42	46	0.43	4.39	1.81	−3.13
Anterior insula - short insular gyri	−38	−4	12	0.68	5.32	4.33	−3.42	38	6	2	0.62	5.39	4.83	−1.73
Posterior insula	−34	−22	12	0.88	5.2	5.26	−2.7	36	−8	−6	0.97	4.75	4.09	−3.02
Anterior segment of the circular sulcus of the insula	−26	20	−8	0.4	4.15	2.67	−2.1	32	24	4	0.45	3.84	4.44	3.35
Inferior segment of the circular sulcus of the insula	−36	−20	4	1.33	6.39	5.11	−2.65	38	−18	0	1.17	5.5	3.6	−1.52
Superior segment of the circular sulcus of the insula	−38	−4	20	0.79	5.73	4.05	−1.39	36	6	8	0.62	5.58	4.72	−0.99
**Temporal Regions**														
Heschl’s gyrus	−40	−22	6	1.62	6.26	5.41	−0.4	40	−22	6	1.23	5.32	4.99	2.92
Transverse temporal sulcus	−48	−24	6	1.48	5.69	5.87	1.62	50	−28	10	1.33	5.68	5.57	3.19
Lateral superior temporal gyrus	−64	−40	12	2.14	5.64	4.8	3.33	62	−20	6	1.9	5.6	4.96	2.79
Superior temporal gyrus - planum polare	−50	−8	0	1.23	4.82	4.41	1.22	50	−6	−6	1.08	4.3	3.75	−0.01
Superior temporal gyrus - planum temporale	−54	−32	12	1.92	5.52	5.39	0.95	60	−24	6	1.55	5.89	4.64	2.78
Superior temporal sulcus	−44	−48	14	1.04	3.83	2.53	0.59	46	−42	14	1.15	6.09	2.94	1.84
Middle temporal gyrus	−62	−54	6	0.88	3.5	2.37	−0.03	60	−58	4	1.18	4.21	2.97	−1.89
Inferior temporal gyrus	−56	−54	−10	0.87	3.41	−1.24	−3.29	58	−44	−12	1.07	3.57	0.66	−2.94
Inferior temporal sulcus	−54	−38	−14	0.6	3.22	0.22	−3.02	56	−42	−8	0.57	3.6	0.72	−2.36
Medial occipito-temporal gyrus - parahippocampal gyrus	−16	−36	−6	1.04	5.17	2.7	−2.49	16	−38	−4	1.11	4.47	2.89	−2.31
**Parietal Regions**														
Superior parietal lobule	−8	−74	44	1.43	4.54	4.05	−1.54	16	−72	46	0.68	3.49	1.76	−1.51
Inferior parietal - supramarginal gyrus	−62	−30	22	1.43	6.43	4.69	−1.37	64	−36	24	1.49	5.24	3.09	0.82
Inferior parietal - angular gyrus	−42	−56	42	0.67	4.31	3.07	−1.42	58	−48	32	0.67	4.97	3	0.02
Sulcus intermedius primus of Jensen	−52	−50	32	0.49	4.51	2.77	−2.66	54	−42	38	0.9	4.77	4.09	0.58
intraparietal sulcus and transverse parietal sulci	−38	−54	40	0.58	4.39	4.77	−0.23	40	−44	38	0.56	3.95	3.67	0.23
Postcentral gyrus	−50	−24	54	2.15	5.2	3.47	−2.66							
Postcentral sulcus	−44	−28	48	0.8	5.47	4.85	−3.78	40	−42	36	0.48	3.65	3.48	−0.13
Paracentral lobule and sulcus	−4	−14	72	1.45	3.06	2.4	−1.93							
Subparietal sulcus	−8	−46	44	0.4	4.06	−0.19	−3.66	10	−58	42	0.43	4.36	0.95	−3.1
Precuneus	−6	−32	44	1	4.93	2.93	−3.86	10	−58	42	0.77	4.36	0.95	−3.1
**Occipital Regions**														
Occipital pole	−8	−94	−4	0.67	4.32	2.73	−1.99	8	−94	14	1.19	3.88	−0.57	−3.45
Calcarine sulcus	−18	−72	4	0.86	4.53	0.93	−2.62	16	−72	10	0.87	4.22	2.52	−2.45
Superior occipital gyrus	−16	−78	38	0.59	2.82	0.66	−2.37							
Cuneus	−6	−78	32	0.92	4.03	3.02	−2.99	10	−78	12	0.98	4.26	1.68	−2.4
Medial occipito-temporal gyrus - lingual gyrus	−6	−94	−6	1.03	4.31	1.91	−2.29	6	−82	0	1.15	4.36	2.6	−2.77
Medial occipito-temporal sculcus and lingual sulcus	−24	−72	−4	0.53	3.2	0.49	−2.15							
Lateral occipito-temporal sulcus	−48	−46	−14	0.38	3.1	−0.53	−3.34							
**Interlobar Regions**														
Horizontal ramus of the anterior segment of the lateral fissure	−32	32	4	0.22	3.76	3.15	−0.61	36	32	6	0.39	4.17	3	−1.22
Vertical ramus of the anterior segment of the lateral sulcus	−36	26	10	0.26	3.31	5.39	0.65	38	22	10	0.27	4.27	5.32	2.8
Posterior ramus of the lateral sulcus	−32	−28	22	1.42	5.72	3.92	−1.94	42	−32	14	1.11	6.55	4.67	1.54
Anterior transverse collateral sulcus	−38	−14	−22	0.49	2.92	1.34	−1.82							
Posteror transverse collateral sulcus	−20	−74	−4	0.3	3.29	−0.25	−2.93							
Parieto-occipital sulcus	−16	−70	36	0.64	4.16	1.13	−1.91	12	−66	34	0.71	4.17	4.1	1.57
Pericallosal sulcus	−4	−34	26	0.97	5.5	4.67	−1.5	4	−30	28	0.83	5.31	4.85	−1.17

Note: x,y,z coordinates in units of mm; PSC: percent signal change of the group average of each subject’s maximum percent signal change in the region; Zmax: the Z-score of the peak voxel of activation within the local cluster; Zc1b: the Z-score of the 1^st^ condition cue+target+standards vs. baseline; Zc2b: the Z-score of the 2^nd^ condition cue+standards vs. baseline.

#### Cue+standards vs. standards only

We first compared activations between the condition where the cue occurred in one of the ears (atop dichotic standard tones) but no target followed, and the condition consisting of standard tones only (**Figure 2A**). This contrast, presumably reflecting cued voluntary attention shifting, was associated with significantly (P<0.05, cluster threshold corrected for the family-wise error based on the theory of Gaussian random fields, GRF) increased activations in the bilateral precentral areas (including premotor cortex, PMC and FEF), anterior insula, medial superior frontal cortex (mSFC) including pre-SMA extending to paracingulate and anterior mid-cingulate cortex (aMCC), dorsal posterior cingulate (dPCC), posterior superior temporal gyrus (pSTG), planum temporale (PT), superior temporal sulcus (STS), angular gyrus (AG), and IPS. Lateralized activations were found in the right inferior parietal region (including supramarginal gyrus, SMG, and the sulcus intermedius primus of Jensen) and left cerebellum. Several subcortical structures including the thalamus, putamen, and caudate were also activated bilaterally.

#### Cue+novel+standards vs. cue+standards

In the second comparison, we contrasted the condition where an unexpected “novel” sound occurred opposite to the cued ear with the condition consisting of the cue and standard tones but no target (**Figure 2B**). This contrast, presumably reflecting novelty-triggered involuntary attention shifting, was associated with significant (P<0.05, cluster threshold corrected for the family-wise error based on the GRF theory) activations in several frontal and cingular cortex regions, including the right PMC/FEF, middle frontal cortex (MFC), and pars triangularis of IFC, as well as in the orbital regions, pregenual ACC, posterior MCC (pMCC), dPCC, and subparietal sulcus regions (i.e., parietal continuum of cingulate sulcus). Activations associated with novelty-triggered involuntary attention shifting were also found bilaterally in the posterior insula, the temporo-parietal junction (TPJ), in the SMG and the AG of inferior parietal regions, IPS, and precuneus. In the temporal lobe, activations associated with this contrast extended to primary (medial 2/3 of Heschl’s gyrus) and non-primary (anterior and posterior STG, PT, lateral 1/3 of Heschl’s gyrus) auditory cortex areas, as well as to the STS and middle and inferior temporal areas. Finally, in this contrast, we also observed activations in the visual cortex (left cuneus) and in several subcortical regions, including the bilateral thalamus and putamen, as well as in the right cerebellum.

#### Cue+target+standards vs. cue+standards

**Figure 2C** shows data from the contrast that compared activations in the condition where the target occurred in the cued ear, to the condition consisting of the cue and standard tones only. In this contrast, presumably reflecting target discrimination, we observed significantly (P<0.05, cluster threshold corrected for the family-wise error based on the GRF theory) increased activations in several frontal, parietal, temporal, and occipital regions. Frontocingularly, activations were found in the bilateral superior frontal cortex, the dorsolateral prefrontal cortex (DLPFC), PMC, IFC, ACC (orbital, subgenual, pregenual), aMCC, pMCC, dPCC, and the pars marginalis. In and near the parietal cortex, the activations extended to TPJ, SPL, the SMG and AG of inferior parietal regions, IPS, the subparietal area, the precuneus, and to the parieto-occipital sulcus. Increased activations to target discrimination emerged also in visual cortices including the cuneus, calcarine sulcus, and lingual gyrus. In temporal areas, in addition to the primary and non-primary (anterior and posterior STG, PT) auditory cortex, target discrimination also activated STS, and the middle and inferior temporal areas. Activations lateralized only to one hemisphere were found in the left central sulcus, postcentral gyrus, and SMA of the mSFC (extending to the paracentral lobule), as well as in right FEF and pre-SMA of the mSFC. Finally, bilateral activations were observed in subcortical regions including the thalamus, putamen, caudate, and pallidum, as well as the cerebellum.

#### Standards only vs. fixation

To examine the demands of standard sounds on attention, we contrasted the condition in which only standard sounds occurred to the condition of fixation (**Figure 2D** **)**. Significantly (P<0.05, cluster threshold corrected for the family-wise error based on the GRF theory) increased activations were observed in several frontal, temporal and parietal regions, including bilateral SFC, primary auditory cortex, posterior insula, STG, paracentral region, and suborbital sulcus. Left lateralized activations were found in MFC, STS, inferior parietal region (AG), while right lateralized activations were found in the central sulcus extending to pre-central and post-central gyrus.

#### Comparisons between activations

**Figure 3:**
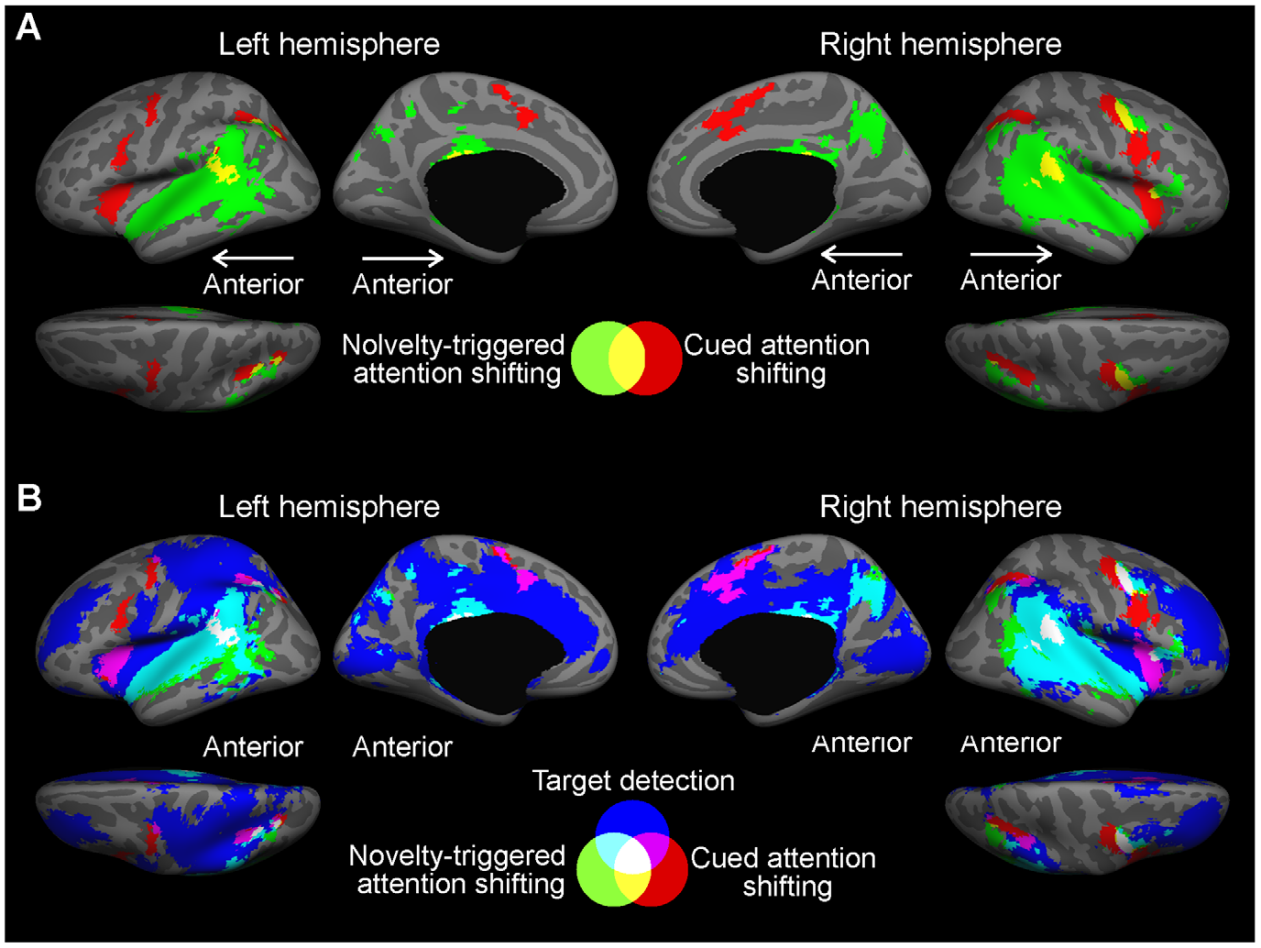
Anatomical labeling of significant activations during the different task conditions based on group (*N* = 18) results. **A.** Comparison between cued attention shifting (Cue+Standards *vs.* Standards Only) and novelty-triggered attention shifting (Cue+Novel+Standards *vs*. Cue+Standards) conditions would seem to support a distinction between a dorsal voluntary (pSTG, PT, STS, anterior insula, superior IPS, IFC, FEF/PMC, mSFC, aMCC) and a more ventral (primary and non-primary auditory cortex, TPJ, inferior IPS, precuneus, PCC, right IFC) involuntary attention system. **B.** When also overlapped with areas activated during target discrimination (Cue+Target+Standards *vs*. Cue+Standards), areas purely related to voluntary attention shifting would seem to be focused to the right and left PMC, including the FEF that is also partially activated by the other conditions, and to the superior/posterior aspect of IPS. Involuntary attention shifting (Cue+Novel+Standards *vs*. Cue+Standards) seems to concentrate in the right inferior IPS and posterior STS (MT/MTG) areas. Interestingly, the anterior insula seems to be activated during both conditions needing voluntary attentional control. At the same time, in the auditory cortices, voluntary attention shifting (Cue+Standards *vs.* Standards Only) seems to be restricted to the posterior “where” area, while both target discrimination and involuntary orienting to novel sounds activated virtually all superior temporal auditory areas.

This analysis was conducted to illustrate and compare areas specifically concentrating on cued vs. novelty-triggered attention shifting, and target discrimination (**Figure 3**). The more limited comparison in **Figure 3A** shows the areas activated significantly in the cued attention shifting (originally shown in **Figure 2A**) and novelty-triggered attention shifting (originally shown in **Figure 2B**) conditions. This comparison, which did not consider the target-discrimination related activations, showed overall distribution differences between areas activated during cued (red color) vs. novelty-triggered (green) attention shifting that are principally consistent with previous models [1]–[4] distinguishing between separate dorsal and ventral attention systems (for anatomical details, see Tables 1 and 2). However, the anterior insula, an area that has been previously often associated with the ventral stimulus-driven/salience detection network, was activated bilaterally during cued attention shifting, while certain areas in the right IFC were activated during novelty-triggered but not cued attention shifting.

However, when activations during auditory target discrimination were also considered (dark blue in **Figure 3B** **)**, the presumed dorsal *vs*. ventral distinction between goal-driven (cued shifting, target discrimination) and stimulus-driven activation networks became slightly less obvious. That is, particularly in the right hemisphere, many of the more “ventral” areas, specifically near the superior temporal auditory areas and in the lower parts of the lateral temporal cortex (STS), which were significantly activated during novelty-triggered (but not cued attention shifting) were also strongly activated during detection of auditory targets. Nonetheless, the more posterior aspects of STS, more extensively in the left hemisphere, seemed to be quite selectively related to stimulus-driven processes (green in **Figure 3B**).

Note that there were also overlaps between the two more goal-driven auditory attention conditions (cued attention shifting and target discrimination) in areas not activated by novelty-triggered attention shifting (pink in **Figure 3B**). One such area is, interestingly, the anterior insula. Overlapping activations between the two more goal-driven auditory attention conditions were also observed in parts of the PMC/FEF and cingulate cortex.

Despite the complex pattern of overlapping activations to the three major contrasts of interest, we also found areas that were significantly associated by only one of the three processes. An interesting distribution of activations was observed particularly in the right posterior parietal areas: cued attention shifting activated the superior and anterior region of IPS, novelty-triggered attention shifting activated the posterior and inferior IPS, while target discrimination activated more anterior/superior aspects of IPS. Finally, areas activated selectively by voluntary attention shifting were also found in the right and left precentral areas, in the vicinity of FEF.

**Figure 3B** show activations in a widespread array of regions related selectively to target discrimination more dorsally and also medially in the neocortex. For example, the superior lateral PFC areas (including DLPFC), the medial PFC, and cingulate cortex areas were activated by target discrimination only (see **Figure 2C** and **Table 3** for detailed anatomical descriptions). Similarly, activations of visual cortex areas, including the calcarine sulcus, cuneus, and lingual gyrus, were almost specific to target discrimination, with only a few activation points found more dorsally near the parietal-occipital junction and cuneus during novelty-triggered attention shifting.

#### Cued attention shifting vs. novelty-triggered attention shifting

**Figure 4:**
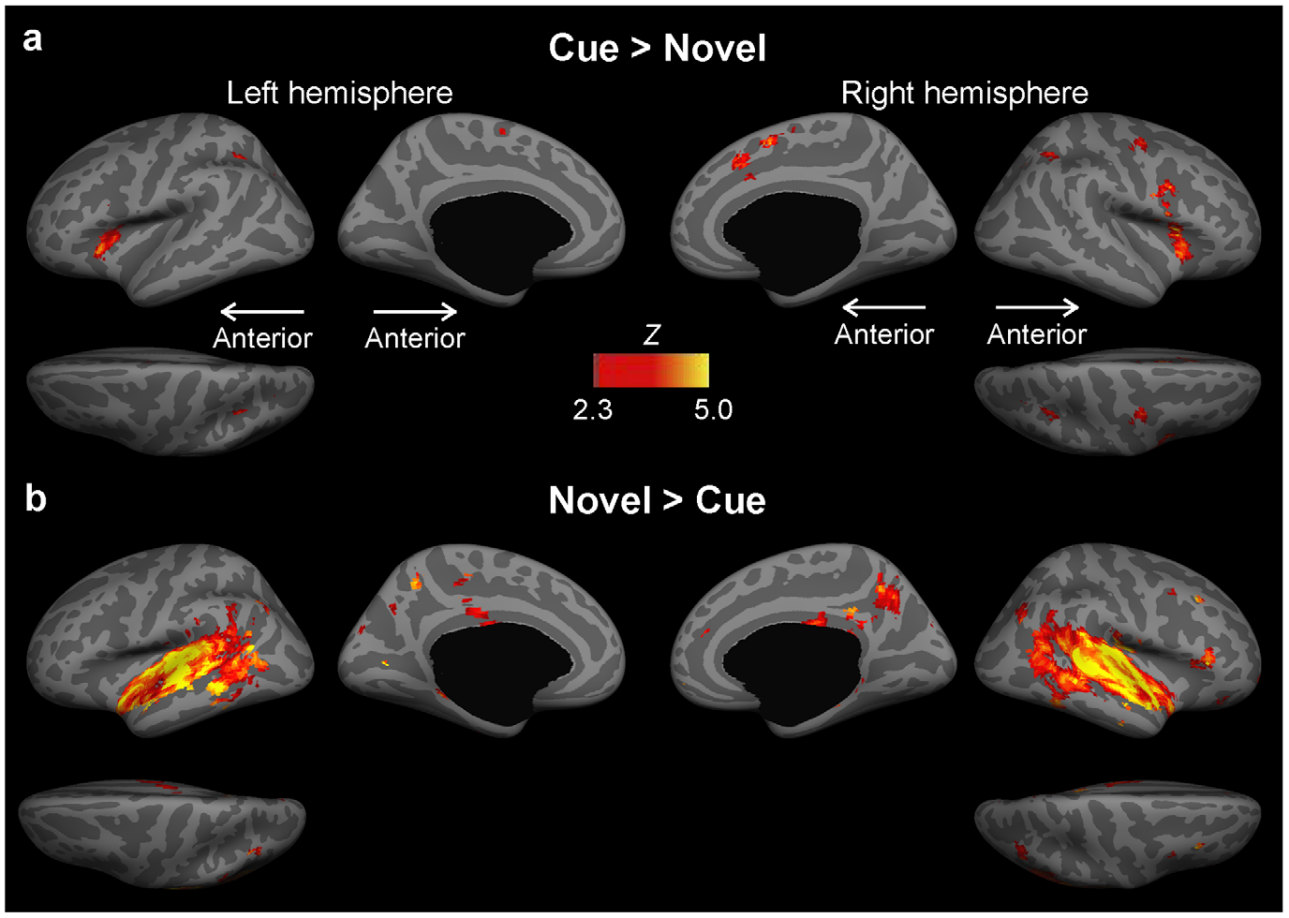
Areas Activated by the Cue vs. Novel and Novel vs. Cue Contrasts. Increased activations associated with Cue vs. Novel contrast were observed in bilateral FEF, mSFC/aMCC, anterior insula, and the anterior/superior aspect of IPS, as well as right PMC and IFC. Increased activations associated with Novel vs. Cue contrast were most prominently in the bilateral primary and non-primary auditory cortex, TPJ, and the inferior aspect of IPS. The contrasts were calculated at the second-level using a random-effects group analysis thresholded at *P*<0.01.

Additionally, we also directly compared cue vs. novel and novel vs. cue contrasts using second-level random-effects group analysis thresholded at P<0.01 (**Figure 4**). Activations associated with the Cue vs. Novel contrast were significantly higher in bilateral mSFC/aMCC (both more prominently at the right), anterior insula, and IPS, as well as right FEF, PMC, and IFC (pars opercularis). Activations associated with the Novel vs. Cue contrast were significantly higher in bilateral primary and non-primary (anterior and posterior STG, PT) auditory cortex, posterior insula, STS, MTG, ITG, TPJ, inferior parietal (SMG, AG), and precuneus, as well as right IFC (pars triangularis).

## Discussion

While a small number of fMRI studies [11], [12], [36] utilizing continuous fMRI scanning on cued auditory attention shifting have been published, the current study was specifically designed to compare auditory fMRI activations between attention shifting to predictable cues and stimulus-driven orienting to unexpected novel sounds. Noting the essential role of sensory areas in automatic change detection processes triggering involuntary attention in the auditory domain, we used a “mixed” event-related/sparse sampling approach to mitigate both sensory and attentional confounds caused by acoustical scanner noise to achieve this goal. In addition to similarities between activations to putative endogenous and exogenous processes consistent with previous studies [7], [9]–[11], our approach also revealed some notable activation differences between cued attention shifting, novelty-triggered attention shifting, and target discrimination.

Areas activated selectively by cued attention shifting were found in the bilateral precentral/FEF regions, and in posterior parietal areas. In these areas, the foci of activations were clearly different between cued attention shifting (more posteriorly/superiorly in IPS), novelty-triggered attention shifting (inferior/posterior to IPS), and target discrimination (more anteriorly in IPS/SMG). In line with the theory [1]–[4] of distinct dorsal vs. ventral attention systems, novelty-triggered attention shifting activated selectively posterior aspects of the STS/medial lateral temporal cortex, inferiorly to the activations of the two other more goal-directed attention conditions. Interestingly, the anterior insula, which has often been associated with more fundamental processes of stimulus-driven change [44] and salience detection [45], [46], was activated during cued attention shifting and target discrimination, but not during attention shifting to sound novelty. In the prefrontal cortices, activations associated with target discrimination were widespread and found in regions more anterior and superior to the frontal areas activated by other conditions.

In the precentral areas including the FEF and lateral PMC areas (BA 6 and 44), most widespread activations were observed for the contrast associated with cued attention shifting. Within these areas, the region probably most closely corresponding to FEF was significantly activated also during novelty-triggered attention shifting, but only very weakly during target discrimination. Our results, thus, would suggest that these areas are related to the orienting of auditory attention, and most strongly, during cued attention shifting. This interpretation is consistent with the long-held view that FEF constitutes a critical locus for the control of spatial attention [47], as it is presumably interconnected with other frontoparietal regions, such as IPS, and because it may also be involved in multisensory attention [48] and orienting [9], [49]. The increased FEF activations associated with auditory attention shifting, during a condition in which subjects were instructed to fixate on a cross in the center of the screen for all tasks, is also in line with the notion [49], [50] that this area is involved in more than just the control of eye movements and overt gaze orienting. However, similar to previous observations [7], [9], [11], our results suggest that the right FEF is activated by distracting events that catch attention in a bottom-up manner as well. Indeed, it was recently suggested that regions of human FEF and IPS may reflect the representation or integration of attentional priority [9], [51], instead of constituting strictly a “voluntary” attentional system. In other words, the function of FEF may, instead of voluntary control only, be more essentially associated with orienting of spatial attention. Intriguingly, the lateralization of present FEF effects for novel and distracting auditory events is also in line with the traditional view that the triggering of involuntary auditory attention is specifically lateralized to the right frontal cortex [40]. Meanwhile, the voluntary shifting condition seemed to activate precentral areas including the FEF more bilaterally.

Our results on precentral areas (beyond FEF) may be interesting in light of the recent debate on the attentional role of lateral PMC. Some studies [2], [3] suggest that lateral PMC is involved in the detection of salient and behaviorally relevant stimuli, especially in unattended and task-irrelevant locations (stimulus-driven attention). This finding has led to a proposition that these regions constitute a part of the same ventral fronto-parietal network that also includes the anterior insula and TPJ [2], [3]. However, the present evidence of more widespread activations in the lateral PMC during cued rather than novelty-triggered attention shifting suggests that these regions are involved in top-down/voluntary attentional control.

The posterior parietal cortex, especially IPS, is essential for spatial attention [32], [52], [53] supported by converging evidence from monkey physiology and human neuroimaging studies. There is even some evidence that further suggests a topographic organization of spatial attention signals within IPS [54]. Our findings that cued attention shifting activates superior IPS, novelty-triggered attention shifting activates inferior IPS, and target discrimination activates more anterior/superior IPS areas suggest possible functional differentiation within these posterior parietal areas. These findings are also principally in line with the proposed dorsal *vs*. ventral distribution of networks dedicated to goal-driven (cued attention shifting, target discrimination) and stimulus-driven (novelty-triggered attention shifting) attentional processes [1], [2], [4].

Another intriguing finding in our study is that the anterior insula was, in both hemispheres, more significantly activated in cued attention shifting and target discrimination than novelty-triggered attention shifting. Previous studies on executive control of auditory spatial attention have reported activations in the bilateral anterior insula [36]. However, the anterior insula has not, traditionally, been viewed as a task-control region, and its activations have typically been considered subsidiary to IFC [11], [33], [55] or they have not been extensively discussed [10], [12]. Nevertheless, our data are in line with the accumulating human and non-human primate evidence [27]–[30], [56], [57] suggesting that the anterior insula, as a part of a cingulo-opercular system, might play a more significant role in voluntary cognitive control than previously assumed. The notion has been further supported by animal [27] and human [58] evidence on anatomical connectivity between the anterior insula and mSFC areas, as well as by histological evidence [59], [60]. It has been, however, also debated whether the anterior insula has a more executive role in maintaining a sustained task mode and strategy [29], or whether it is merely a transient saliency detector that initiates attentional control signals in other higher-order areas [46]. The present lack of anterior insula activations to the most salient sounds of the present design, the novel sounds, would seem to be clearly at odds with the latter idea. Our findings would, instead, seem to be more consistent with an alternative theory [61] that the anterior insula activity does not express perceptual salience, *per se*, but rather the recruitment of processing resources when faced with a given sensory event, whatever the source of that recruitment, bottom-up or top-down. Finally, it is also noteworthy that, in addition to the actual redirection of attention, attention shifting presumably involves endogenous processes that allow us to disengage from previous activity and to maintain heightened top-down control on the new task [62]. Consequently, noting the recent evidence by Wu et al. [36] and Alain et al. [32] showing anterior insula activations during working memory processing and goal-directed actions, it is also possible that activations in the anterior insula during cued attention shifting and target discrimination are most essentially related to engagement of attention control.

It has been proposed that the anterior insula and the cingulate gyrus may belong to the same cingulo-insular system involved in top-down cognitive control. Our data is consistent with this proposal, as the mSFC regions (including bilateral pre-SMA and extending to aMCC, and rostral cingulate zones) and the anterior insula were both activated during cued attention shifting and target discrimination, but not in novelty-triggered attention shifting. The cingulate gyrus has also been identified as a major component in a distributed network subserving the dynamic relocation of spatial attention [63], [64]. Previous studies also show that aMCC is associated with conflict resolution [65], [66] and decision making [67]. Here we provide data suggesting that aMCC is specifically involved in top-down spatial attention control.

In general, our data are consistent with previous studies on auditory attention shifting. However, we also observed certain discrepancies. Such discrepancies may, obviously, be in part explainable by differences in paradigms and methods between the studies. For example, our results differ slightly from a previous event-related fMRI study by Mayer et al. [10] where subjects were instructed to localize targets following informative (75% valid) or uninformative (50% valid) cues. In contrast to present findings, as well as those reported in several recent auditory studies [11], [68], the authors found that automatic orienting elicited by the uninformative cue condition increased activations in the precentral areas and the insula, both of which in the present study were associated with voluntary instead of stimulus driven processes. However, the study of Mayer et al. apparently did not aim at separating the processes of auditory cued attention shifting and target identification. Moreover, based on the behavioral data, it is not entirely clear that uninformative cues used in Mayer et al. were, in fact, followed by less intensive top-down processing than informative cues.

At the same time, the present results differ slightly from the event-related fMRI study of Salmi et al. [11], in which the authors used a dichotic target-discrimination design that was in many ways analogous to the present study. Specifically, Salmi and colleagues asked their subjects to detect occasional targets in the attended stream. Instead of novel sounds presented only to the uncued ear, involuntary attention shifts were triggered by unexpected loudness deviations presented to either ear. Finally, unlike in the present study, attention shifts were guided using central visual cues. Their results were quite similar to the present findings in regards to top-down controlled attention shifting. Salmi et al did not, however, observe top-down related activations in the anterior insula. At the same time, using the visually presented shifting cues, Salmi et al. observed visual-cortex activations that were absent during cued shifting in the present study. For the bottom-up driven attention shifting, the present study showed more extensive activations in bilateral auditory cortex (possibly due to auditory stimulation and scanning parameter differences explicated above), posterior insula, IPS, and posterior cingulate than the study of Salmi and colleagues. In addition to aforementioned differences in stimulation and scanning parameters (continuous vs. sparse sampling), some of these discrepancies may be explainable by anatomical interpretation approaches. For example, the present surface-based approach may produce different results in terms of the exact anatomical boundaries between the anterior insula and IFC than the fully volumetric atlas that was used by Salmi and colleagues.

The present sparse sampling design may help make the results more easily comparable to cognitive neuroscience studies conducted using other methods that are not confounded by factors such as acoustical scanner noise. That is, in comparison to the relatively small number of auditory studies on voluntary attention shifting, there has been a profusion of MEG and EEG research on involuntary attention shifting to unattended sound changes [15]–[17], [69]–[77]. In these studies, involuntary auditory attention shifting has been proposed to be triggered by an automatic change-detection process, reflected by the MMN response [78], followed by a sequence of brain events associated with attentional orienting and conscious detection of this change (however, see also [79]). Indeed, in the present study, quite remarkable differences between conditions emerged in the superior temporal auditory areas. In these areas, the activations during novel sound processing extended all over the superior temporal plane, while activations to attention shifting cues were only significant in posterior aspects of auditory cortex (pSTG, PT). This distribution difference of effects could, in principle, be interpreted to be in line with the suggestion [78] that automatic deviance detection (reflected by the MMN process) originates more anteriorly in the auditory cortex than responses to more predictable shifting cues. At the same time, previous studies also suggest that non-primary auditory cortex processes sound identity and location in parallel, through the anterior “*what*” and posterior “*where*” pathways [52], [80]–[82]. In the current study, the novels contained much richer identity features than the cues used to trigger voluntary attention shifting. Hence, the enhanced spreading of auditory cortex activations to the putative “*what*” regions might reflect stimulus-driven activations in the sound-object identification system. A sound-identification process might also explain some of the IFC activations during novelty-triggered attention shifting, given the theory that the “what” streams extend to ventral frontal cortex areas [81], [82]. Meanwhile, the broader activations associated with auditory target discrimination, compared to cued attention shifting, could be partially explained by the more enhanced top-down influences needed for the more difficult process of discriminating the targets from the repetitive standards, as established by numerous imaging studies [83]–[85]. Our previous work on auditory attention has also demonstrated correlations between attentional modulation of auditory cortex activation and behavioral discrimination of target tones (as measured from the difference in the hit rate between easier vs. more difficult targets delivered to the ear).

We observed extensive activations in the visual cortices associated with target discrimination, similar to Wu et al. [36] (note however that Wu and colleagues asked their subjects to keep their eyes shut throughout the study). This may be the result of cross-modal influences between the auditory and visual cortices. That is, previous studies have shown that the visual cortex can be activated by auditory input [50], [86], [87] and that there are direct anatomical connections between the superior temporal and occipital regions in primates [88] and humans [89], [90]. Meanwhile, a recent fMRI study [91] also showed that auditory occipital activations depend strictly on the sustained engagement of auditory attention and are enhanced in more difficult listening conditions.

The “standards only” condition, during which subjects were instructed to listen carefully and wait for the cue, revealed significantly increased activations in several frontal, temporal and parietal regions. Interestingly, the activated areas included the paracentral region, which according to recent studies is activated during maintenance of attention [92]. These paracentral activations might have also overlapped with a supplementary eye field region, which has been previously proposed to be involved in visuospatial control processes and performance [93]. The areas activated during the standards only condition also included the SFC, which has been previously associated with high-level cognitive control processes such as monitoring [94] and anticipatory spatial attention [95]. However, it has to be noted that, in contrast to other comparisons that were conducted across active task/stimulation conditions, the “standards only” comparison was contrasted with the fixation condition, in which no explicit task was included.

### Benefits of Sparse vs. Continuous Scanning

Here, we utilized sparse sampling to control for biases caused by the acoustical scanner noise. Acoustical scanner noise is potentially a problematic variable in all fMRI experiments, but it is of particular concern in studies of audition and language processing. First, as discussed above, these effects might obviously modulate stimulus-driven orienting, which presumably receives a major contribution from auditory cortex [15]–[17], [69]–[77]. Although scanner noise does not necessarily entirely abolish change-detection activations that trigger involuntary orienting [96], the benefits of sparse sampling in studies on stimulus-driven auditory cortex activities have been well documented [97]–[99]. Second, the ongoing acoustic and somatosensory stimulation associated with continuous scanning may also confound attentional top-down effects, both in auditory cortices and higher-order association areas. The longer-term effects of continuous environmental noise on our ability to concentrate have been very well documented [100]. Not surprisingly, in fMRI studies, it has been shown that increasing the intensity of acoustical scanner noise modulates extra-auditory activations during working memory performance, resulting activation increases in certain areas (inferior, medial, and superior frontal gyri) and decreases in others (*e.g.*, anterior cingulate) [101]. Using PET, it has been further shown that recorded scanner noise may increase regional blood flow in anterior cingulate and Wernicke’s areas during visual imagery [102]. Event-related potential (ERP) studies also suggest that continuous fMRI scanner noise may reduce and delay certain “endogenous” components that are related to auditory attention [96]. Consistent with these results, active behavioral auditory performance may be improved during sparse vs. continuous fMRI scanning [99]. Finally, recent sparse-sampling fMRI studies [103] also suggest that top-down attention effects may produce detectable modulations in auditory cortex even in the absence of any acoustic stimuli. Such endogenous feedback activations could be easily masked or cofounded in by acoustical scanner noise. These kinds of confounds have been also discussed [104], [105], for example, in the context of interpreting top-down modulations of auditory cortex activity by visual stimuli during continuous scanning.

Based on the above notions, it might seem quite obvious that sparse sampling is the best approach for any study involving auditory functions. However, it has to be also noted that with sparse sampling designs, a much smaller number of volumes can be acquired in a given experiment, which may reduce the signal-to-noise ratio in comparison to continuous scanning. Indeed, a recent auditory cortex mapping study [99] (which however also utilized 70 dB noise masking on the background in both fMRI scanning conditions) showed relatively small differences between sparse and continuous scan experiments. In terms of more complex designs, a disadvantage of sparse sampling is the reduced temporal resolution that will make it difficult to extract stimulus specific BOLD time courses. Finally, a trade-off in sparse sampling is the fact that the clustered scan noise may, itself, become a “rare sound” that triggers strong activations of the alerting and orienting networks. Although the BOLD responses of such activations will not be necessarily caught by fMRI when the TR is long enough, the cognitive significance and relative saliency of the subsequent stimuli of interest may still be modulated. However, a novel feature in the present orienting design was that these biases were controlled by using the noise stimulus produced by each fMRI volume acquisition as a part of the task design.

### Potential Limitations

It is noteworthy that in experimental conditions, it is difficult to produce and document activations that are purely stimulus driven vs. endogenous. For example, novelty-triggered attention shifting may involve a number of top-down processes that are associated with, for example, the suppression of involuntary attention shifting, reorienting to the relevant task (if this is part of the instruction), and conflict resolution processes to “evaluate the situation” after the automatic orienting response (see, e.g., Escera et al. [16], Schröger and Wolff [41] ). Cued attention shifting is, in turn, contaminated by stimulus-driven processes triggered by the cue itself. At the same time, voluntary attention shifting may involve active disengagement from the previous strategy, as well as engagement to the new attentional task (termed “cued attention,” in Petkov et al. [106], also see [99]). In other words, although the process is collectively referred to as “attention shifting”, the parts that are actually the most “voluntary” or “goal directed/endogenous” might not be related to the orienting, *per se*.

It is also possible that differences in auditory cortex activities during the triggering of involuntary and voluntary attention are related to the context and predictability of the stimulation. Strong unpredictable stimuli, such as novel sounds, tend to result in widespread sensory responses from the bottom up, which then triggers an involuntary orienting process that, according to previous ERP studies, is related to the strength of the auditory cortex response. A more predicable and repeated stimulus, such as the cue, may trigger less prominent responses – but even in this case, the cue plays a role in orienting. However, proportionally speaking, the bottom-up influence is smaller than in the case of novelty-triggered attention (consistent with our predictions and conclusions). At the same time, these processes are essentially modulated by top-down attention, especially when the discrimination task is difficult (such as in the case of targets that result in a stronger auditory cortex response than the cues).

Also note that in most visual studies, the cue that triggers voluntary shifting is usually an arrow, which is physically different from the target. A related consideration is whether the present cues were more prone to induce stimulus-driven activations than the symbolic arrows, which have been utilized in many visual and also auditory attention shifting studies [11]. It has been thought that because arrow symbols do not occur in the physical location of the target, they might trigger purely goal-driven processes. However, it is worth noting that the processing of any simple cue probably gets rapidly automatized during the course of an experiment, and as the simple symbol is repeated, the account of stimulus-driven processes subsequently increases [107], [108]. Most importantly, the present study showed notable differences between activations during cued and novelty-triggered auditory attention shifting, clearly beyond areas associated with sensory cortices processing physical properties of the sounds. Further, activations in these sensory areas, where one might expect particularly strong stimulus-driven activations, were clearly weaker during attention-shifting cues than during novel-sound or target-sound detection.

### Conclusions

In conclusion, our study revealed distinct activations during cued auditory attention shifting, involuntary orienting to novel sound, and auditory target discrimination. Areas most selectively involved with cued voluntary shifting included the superior/posterior IPS and precentral areas (including FEF and PMC), which provides important evidence supporting these regions’ involvement in top-down/voluntary attentional control. Activations specific to involuntary attention shifting to novel sound were found in posterior STS, inferior IPS and TPJ, which is principally consistent with models suggesting more ventral distribution of stimulus-driven attention [1], [3], [4], [109], but also from the right IFC. Interestingly, our results also revealed marked differences in the anterior insula and IFC activations associated with goal-driven attentional processing (cued attention shifting and target discrimination) and novelty-triggered involuntary attention shifting, suggesting that the anterior insula may play a more executive role in auditory attention than previously thought.

## Materials and Methods

### Participants

Potential subjects were first screened with a phone interview to ensure that they had normal hearing and had not been exposed regularly to environments with excessively loud noise. Nineteen right-handed college-level educated adults with normal hearing and no neurological disorders, psychiatric conditions, or learning disabilities, gave written informed consent prior to testing, in accordance with the experimental protocol approved by the MGH IRB. One subject was excluded from the final sample due to an inability to perform the task (hit rate below 50%), rendering a total of eighteen subjects (N = 18, 11 females, age range 19–28).

### Cued Auditory Attention Shifting Task

In all trials, brief pure tones (duration 50 ms, 5-ms ramps) were presented in the background, randomly to the right (800 Hz) or left ear (1500 Hz), similar to a classic study [39]. Because the standard sounds merely offered a context to other sounds (which were consistent across ears), the ear/frequency order of the background standard stream was held constant across subjects. Subjects were told to wait for a cue (250-ms buzzer sound) that occurred in the ear where a subsequent target (50-ms tone with 800- and 1500-Hz harmonics) was likely to occur. The average interval between the cue and the target was ∼1.7 sec. Upon hearing the cue, the subjects were advised to shift their attention to the designated ear (with eyes remaining fixated), pay close attention to the tones presented in that ear, and press a button with the right index finger as rapidly as possible after hearing the target. Specifically, subjects were instructed to pay attention to a change in relation to the ongoing stimulation (a “thickening” of the sound), and they were kept naïve to the fact that the targets were actually similar in both ear streams.

Previous event-related MEG/EEG studies [15], [21], [110] suggest that strong event-related MEG/EEG responses (*e.g.*, the P3a component) associated with involuntary auditory orienting can be evoked by physically varying “novel” sounds. In 20% of the trials, the target was therefore replaced by a task-irrelevant novel sound presented opposite to the cued ear. These novel sounds consisted of eight spectrotemporally complex environmental and synthetic sounds whose peak intensities, onset rise times, and perceived loudness, as well as their grand-average time envelope, were made as close to the cues as possible. Pure tones only (no cue, novel, or target) were presented in 20% of trials. At 7.82 sec after the trial onset, subjects heard the sound of 2.18-sec fMRI volume acquisition signaling that the trial had ended. In other words, the confounding effects of fMRI acquisition noise were controlled by using it as a task stimulus. Tonal stimulation started 2.3 sec after the onset of preceding scan/simulation, at a 1.1-sec average stimulus-onset asynchrony (SOA) in each ear, and ended on average 1.3 sec before the next scan. The SOA was jittered within each trial to avoid omission-response confounds. During fMRI, three silent baseline trials occurred after every 6 active trials (i.e., a mixed blocked/event-related design was utilized). In subsequent analyses, individual trials with target-detection responses beyond the subject’s mean ±2SD reaction time were considered outliers. Finally, in an additional ten-minute behavioral experiment testing whether the spatial cueing indeed produced significant performance benefits, we replaced 50% of the novel sounds with a target sound opposite to the cued ear (“invalidly cued target”).

### Procedure

A standardized computerized approach taking about 5 minutes was utilized to teach the task to the subjects before scanning. During fMRI sessions, subjects were presented with randomly ordered 10-sec trials. Sound stimuli were presented at 55 dB Sensation Level, as tested individually at the beginning of each session, and delivered through MRI compatible insert earphones (Sensimetrics, Malden, MA). The insert included an eartip to protect the subjects’ ears during the scan acquisitions. A cross (fixation mark) was projected on the center of an MRI compatible video display. Subjects were instructed to look at the fixation mark throughout the whole study. Each scan session contained three runs, and there was a brief break after each run to restart the stimulation and communicate with the subject. For each task run, there were 136 trials/blocks that lasted 22 minutes and 40 seconds. Subjects were instructed to respond with their right index finger.

### Data Acquisition

Whole-head fMRI was acquired at 3T using a 32-channel coil (Siemens TimTrio, Erlagen, Germany) and an interleaved echo planar imaging (EPI) method. To circumvent response contamination by scanner noise, we used a sparse-sampling gradient-echo blood oxygen level dependent (BOLD) sequence (TR = 10 sec, TE = 30 ms, 7.82 sec silent period between acquisitions, flip angle 90°, FOV 192 mm) with 36 axial slices aligned along the anterior-posterior commissure line (3-mm slices, 0.75-mm gap, 3×3 mm^2^ in-plane resolution), with the coolant pump switched off. A field mapping sequence (TR = 500 ms, flip angle 55°; TE1 = 2.83 ms, TE2 = 5.29 ms) with the same number of slices, voxel size, and slice orientation to the EPI sequence was applied to obtain phase and magnitude maps utilized for unwarping of B_0_ distortions of the functional data. T1-weighted anatomical images were obtained for combining anatomical and functional data using a multi-echo MPRAGE pulse sequence (TR = 2510 ms; 4 echoes with TEs = 1.64 ms, 3.5 ms, 5.36 ms, 7.22 ms; 176 sagittal slices with 1×1×1 mm^3^ voxels, 256×256 mm^2^ matrix; flip angle = 7°).

### Data Analysis

The fMRI data were preprocessed using tools from FEAT Version 5.98, a part of the FSL package [111] (www.fmrib.ox.ac.uk/fsl). Skull stripping was performed with BET, B_0_ unwarping using FUGUE, and motion correction with MCFLIRT. The data were smoothed with a Gaussian kernel (5-mm FWHM) and registered to the Montreal Neurological Institute (MNI) space using FLIRT. The intensity normalized fMRI time-series were then entered into a general linear model (GLM) with the task conditions as explanatory variables. At the second-stage, individual experimental runs were combined within each subject by using a fixed-effect model. Finally, contrasts pertaining to the main effects of the factorial design constituted the data for the third-stage (mixed-effect) analysis with automatic outlier detection [112], where the significance of observations was determined across the group of 18 subjects using FMRIB’s Local Analysis of Mixed Effects (FLAME) 1 and 2 [111], [113],

Group analysis was performed in MNI space. Gray-matter partial volume information obtained from each subject using Freesurfer 5.0 anatomical segmentation results was entered as voxel-dependent anatomical covariate in the group statistics [114]. The *Z*-statistic images were corrected for multiple comparisons using whole-brain cluster correction based on GRF theory, with an initial cluster threshold of *Z*>2.3 and a post-hoc corrected threshold of *P*<0.05 [115]. Finally, to interpret the anatomical results, the results were coregistered to the FreeSurfer brain template (“fsaverage”) and shown in the surface space. The contrasts presumed to reflect cued attention shifting, novelty triggered reorienting, and target discrimination processes were defined as “cue + standards *vs*. standards only,” “cue + novel + standards *vs*. cue + standards,” “cue + target + standards *vs*. cue + standards,” respectively. Additionally, the “baseline” contrast, *i.e.* “standards vs. fixation” was calculated to examine the effect of standard sounds on attention. Finally, cued attention shifting and novelty-triggered attention shifting were directly compared by defining the “cue *vs*. novel” and “novel *vs*. cue” contrasts at the second-level using a random-effects model of the group analysis with a threshold at *P*<0.01. Finally, behavioral results were analyzed using paired and independent-samples t-tests as appropriate.
